# Experimental models of pancreas cancer: what has been the impact for precision medicine?

**DOI:** 10.1172/JCI191945

**Published:** 2025-08-15

**Authors:** Vasiliki Pantazopoulou, Casie S. Kubota, Satoshi Ogawa, Kevin Christian Montecillo Gulay, Xiaoxue Lin, Hyemin Song, Jonathan R. Weitz, Hervé Tiriac, Andrew M. Lowy, Dannielle D. Engle

**Affiliations:** 1 Salk Institute for Biological Studies, San Diego, California, USA.; 2 Department of Clinical Science, Intervention and Technology, Karolinska Institute, Stockholm, Sweden.; 3 Department of Surgery, Division of Surgical Oncology, Moores Cancer Center, UCSD, San Diego, California, USA.

## Abstract

Pancreatic cancer has a 5-year survival rate of approximately 13% and is projected to become the second-leading cause of cancer-related deaths by 2040. Despite advances in preclinical research, clinical translation remains challenging, and combination chemotherapy remains the standard of care. The intrinsic heterogeneity of pancreas cancer underscores the potential of precision medicine approaches to improve patient outcomes. However, clinical implementation faces substantial challenges, including patient performance status, metastatic disease at diagnosis, intrinsic drug resistance, and a highly complex tumor microenvironment. Emerging targeted therapies, such as RAS inhibitors, offer promise for personalized treatment. These developments have prompted precision medicine–focused clinical trials using molecular subtyping for patient stratification. Effective development of precision medicine therapies depends heavily on robust preclinical models capable of accurately recapitulating the complexities of the pancreatic tumor microenvironment. Two-dimensional, air-liquid interface, and patient-derived organoid cultures combined with in vivo genetically engineered mouse models and patient-derived xenografts represent valuable experimental systems. This Review critically examines the strengths and limitations of these experimental model systems. We highlight their relevance and utility for advancing precision medicine strategies in pancreas cancer.

## Introduction

Pancreas cancer is projected to become the second-leading cause of cancer-related deaths by 2040 (1) and has a 5-year survival rate of only 13% (2). It has been historically difficult to translate promising preclinical studies into improvements in patient survival, and the standard-of-care treatment remains combination chemotherapy. Given the heterogeneity of pancreas cancer, precision medicine presents an alternative that could lead to higher patient survival rates.

The complexity of the biology of pancreas cancer has made it notoriously difficult to treat, as covered in several comprehensive reviews (3–6). Curative treatment of patients with pancreas cancer requires surgery, but up to 85% of patients are ineligible owing to spread of the disease at diagnosis (7). Patients with inoperable pancreas cancer typically receive chemotherapy, and those with locally advanced disease may also be treated with radiation (7). Unfortunately, all treatment strategies are associated with debilitating, and sometimes permanent, dose-limiting toxicities, often due to the inherent toxicity of the treatment or the poor performance status of the patients (8, 9).

Different molecular subtypes of pancreas cancer respond differently to therapies (10–13). This heterogeneity in treatment responses warrants the use of precision medicine for the treatment of patients with pancreas cancer. As a result, several clinical trials have been designed to take advantage of molecular subtyping for patient stratification (14). In this new era of novel targeted therapies, such as RAS inhibitors, precision medicine could lead to a paradigm shift in our approach to treat pancreas cancer. Yet precision medicine approaches are challenging given the multifactorial challenges associated with pancreas cancer. These include the patient performance status at diagnosis, systemic comorbidities, distal metastasis at diagnosis, inherent treatment resistance, interpatient heterogeneity, and a complex tumor microenvironment.

Preclinical models have been critical in understanding the biology of pancreas cancer, as well as the often dominant role the extensive tumor microenvironment (TME) plays in treatment response. The nuances of each experimental model system warrant a review of their optimal application and ability to encompass the diverse aspects of pancreas cancer. Here, we discuss the strengths and weaknesses of experimental models, as well as their relevance to precision medicine approaches in pancreas cancer.

## In vitro models

### 2D cell cultures.

Human- or mouse-derived two-dimensional (2D) cell culture models have been extensively used to study pancreas cancer. They are cost-effective and easy to manipulate. Their use in pancreas cancer research has been extensively reviewed elsewhere (15). In short, 2D models have been widely used in genetic perturbation studies (16), transcriptome analyses (17), and high-throughput drug-discovery efforts (18, 19). Preclinical studies using common mouse-derived 2D pancreas cancer cell lines identified that nab-paclitaxel synergizes with gemcitabine (20), a finding that informed a successful clinical trial (21) and has since modified clinical practice (7). Moreover, 2D models have been used to identify mechanisms of resistance to the standard-of-care chemotherapy gemcitabine (22, 23). 2D cell cultures primarily model cancer cell–intrinsic features in the absence of exogenously added extracellular matrix or stromal components. However, cocultures or conditioned medium experiments using 2D cell cultures have successfully modeled the interactions between cancer cells and the desmoplastic and immunosuppressive TME that is characteristic of pancreas cancer (24–26).

While 2D cell cultures are cost-effective and amenable to a wide array of perturbations, they lack 3D architecture, which is necessary for cell-intrinsic and -extrinsic signaling (27). As a result, these models often simplify intricate autocrine and paracrine cell signaling pathways, making predictions of drug responses difficult. Moreover, with the exception of a few immortalized human pancreatic duct epithelial cell lines (28), human primary, non-neoplastic cells are short-lived when cultured in 2D and do not present a good model for long-term experimentation (29). Regardless, 2D patient-derived cell lines present great tools to study the driving oncogenes of pancreas cancer. For instance, they have been extensively used to characterize the efficacy of the novel RAS inhibitors against pancreas cancer (30, 31), as well as to predict mechanisms of resistance to these inhibitors (30, 32).

### 3D models: spheroids, air-liquid interphase, and organoids.

3D cell culture models offer an alternative to 2D cultures. One of the most widely used 3D models are cancer cell spheroids, cell aggregates that are propagated in basement membrane extracts (BMEs) or in attachment-free conditions (33). Pancreas cancer spheroids have been established from different 2D cell lines, including MiaPaCa-2, Panc-1, and Capan-1 (34, 35). Spheroids from these and other pancreas cancer lines express several genes related to epithelial-mesenchymal transition (35, 36) and have been used in high-throughput drug screens (34). Cocultures of pancreas cancer cell spheroids with stromal or immune cells have been used for drug resistance studies and functional analyses (37, 38). The main caveats regarding spheroid models are that they are difficult to maintain, lack polarity, and generally form homogeneous spheres without a lumen (39).

Another 3D in vitro model is the air-liquid interface (ALI) culture (40). Unlike traditional cultures, ALI cultures expose the upper layer of cells to air while the lower compartment remains submerged in medium, creating a physiologically relevant environment for tumor cells, but also for fibroblasts and immune cells (40, 41). These models have been useful for investigating desmoplasia, cancer-associated fibroblast (CAF) interactions, and immune cell infiltration, making them powerful tools in immunotherapy research and for developing TME-targeted therapeutic strategies (42). Despite recapitulating the TME better than 2D cultures or spheroids, the complex tissue architecture of ALI cultures is only preserved during early passages (1–4 passages), with later passages acquiring a cystic morphology (40). This limits the use of ALI cultures for long-term experimentation when the TME is a focus of the study. Further, ALI cultures are difficult to scale for high-throughput approaches, often necessitating conversion into spheroid or organoid approaches for drug testing applications.

Organoids are a 3D model derived from patient or mouse normal or tumor tissues and represent a considerable advancement in pancreas cancer research. Organoids are self-organizing and self-renewing structures, grown in the presence of BME and growth factors (43, 44). Pancreas cancer organoids closely model the genome of their corresponding tumors (45). Interestingly, patient-derived organoids (PDOs) exhibit predominantly a classical-like state, regardless of the state of their corresponding patient tumors (46). This shift is restored when organoids are cultured in the presence of TGF-β ligands (46), highlighting the importance of modeling the TME in in vitro models. The WNT signaling dependency of organoids is also associated with the classical transcriptional subtype, while basal PDOs are more likely to be WNT independent (47). To study the cancer and TME crosstalk, human- and mouse-derived pancreas cancer organoids have been cocultured with either matched or unmatched fibroblasts. These studies have improved our understanding of the pancreas cancer TME, leading to one of the first definitions of functional CAF subtypes (39, 48).

The BME used for the culture of organoids affects the proliferation of organoids but not their drug response or gene expression, emphasizing the robustness and reproducibility of this model system (49). To avoid the lot-to-lot variability of commercially available BMEs, such as Matrigel, PDOs can be cultured in engineered hydrogels with well-defined composition (50, 51). Apart from improving reproducibility, these hydrogels allow for the study of the ECM composition and stiffness (50, 51). However, hydrogels have only been used to test Matrigel-established PDO lines. The generation efficiency or proliferation of PDOs has not been compared between BME and hydrogel-based cultures.

PDOs have emerged as a powerful tool for therapeutic studies. They can be established from patients at all stages of pancreas cancer progression, even from minimal tissue samples such as fine-needle tumor biopsies (52–55). Morphological subtyping of PDOs could facilitate personalized therapy, as cystic gland-like PDOs are associated with the classical transcriptomic subtype, whereas morphologically dense PDOs correspond to a more basal-like subtype, each with distinct drug susceptibilities (56). Work by Tiriac et al. developed a comprehensive framework for testing single-agent chemotherapy and targeted therapies using a large cohort of 66 PDOs (54). Notably, retrospective comparisons to a subset of patient outcomes demonstrated the predictive power of PDOs (54). In a parallel study, 30 PDOs were established and used to screen a library of 76 compounds, revealing novel actionable vulnerabilities (57). To demonstrate clinical utility in a precision medicine assay, PDO-based drug sensitivity in 28 chemo-naive patients was prospectively evaluated, establishing a predictive model for treatment response (58). Pretreated PDOs displayed diminished predictive accuracy compared with chemo-naive PDOs, indicating that prior exposure to therapy may complicate ex vivo drug testing. Moreover, an algorithm has correlated PDO response to patient response (59), further highlighting the predictive value of PDOs.

PDOs present useful tools not only for studying treatment responses but also for predicting the acquisition of resistance. When treatment-naive PDOs were compared with FOLFIRINOX-exposed ones, pretreated models exhibited resistance to irinotecan and oxaliplatin and underwent metabolomic reprogramming (60). The correlation between patient response and PDO drug sensitivity has been confirmed in large cohorts where PDOs accurately reflected responses to neoadjuvant regimens and facilitated rapid drug screening within 7 days of resection (61). Interestingly, this study reports a high PDO generation success rate (over 70%) using both treatment-naive and pretreated surgically resected tumors. Moreover, PDO cultures were used to identify alternative therapies for chemotherapy-resistant PDOs (53) and to uncover the synergy between KRAS and ERBB inhibitors (30). The ORGANOPREDICT trial further underscored the predictive power of PDO-based pharmacotyping in advanced refractory pancreas cancer, identifying effective drugs in most cases, even in heavily pretreated individuals, and validating the synergy between KRAS and ERBB inhibitors (62).

**Figure 1:**
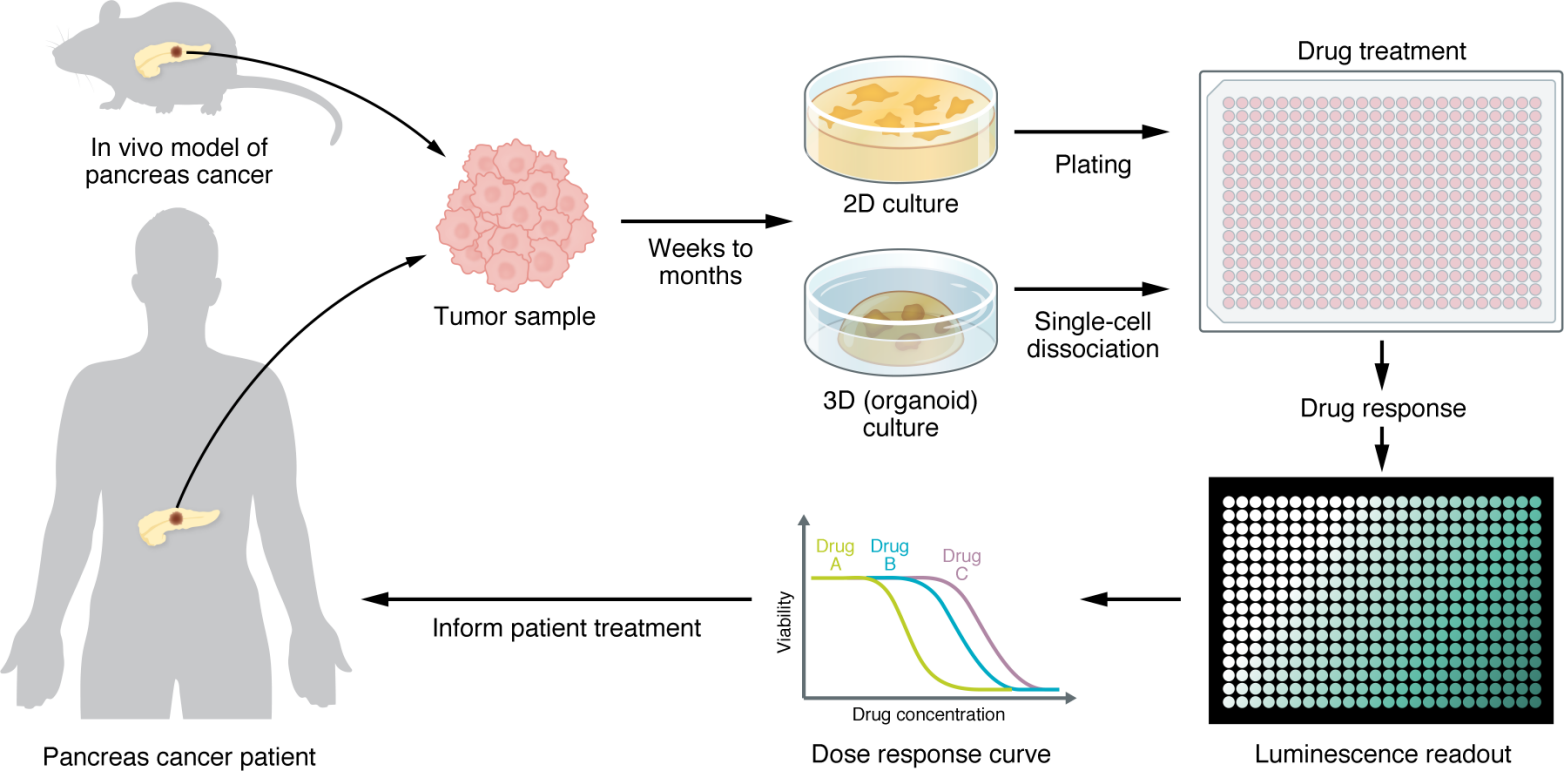
Drug testing pipeline for human- or mouse-derived in vitro models. 3D organoids or 2D cell lines are established from human or mouse pancreas cancer tissue within weeks (for mouse and human samples) to months (for human samples) after resection. After establishment, the cultures are plated in 384-well plate format, treated with the selected drugs using a drug dispenser, and incubated in the presence of the drugs for 3–5 days. The cell viability is assessed using a viability assay, such as CellTiter-Glo (Promega), and a dose-response curve is generated. In a precision medicine pipeline, the most effective drugs are selected for patient treatment.

Pancreas cancer PDO generation rate across these studies is high, and drug screening can typically occur within 2 months after resection for most patients (Figure 1). Several clinical trials, including PREDICT-PACA, ESPAC6/7, and PASS1, are incorporating PDO generation and drug testing to evaluate the concordance between PDOs and the corresponding patients (63). The results of these trials will further highlight the relevance of PDOs in precision medicine approaches.

## Ex vivo models

Ex vivo models, such as slice cultures and tumor-on-a-chip models, have been developed as an orthogonal approach to 3D models.

Ex vivo slice cultures of pancreatic tumors have been established from both murine and human tissues. Ex vivo slice cultures are uniquely situated to examine the pathobiology of the TME as both the cellular and acellular biology is preserved during ex vivo culture. Tumor slice cultures are produced by embedding of resected tissues in low-melting-point agarose and subsequent generation of 200 to 300 μm slices using a vibrating microtome. Tumor slices are cultured in media containing serum, with numerous reports having demonstrated functional studies for up to 1 week after slicing (64–66).

The cellular architecture of tumor slices is maintained during culture, creating an environment permissive to studying functional interactions between tumor cells and multiple TME components, including CAFs, immune cells, and Schwann cells (67–69). Furthermore, translational studies investigating chemotherapeutic and targeted therapies in tumor slices have been useful to determine antitumor responses and changes in the TME during therapy (70, 71). Studies correlating treatment response of ex vivo tissues with patient outcomes will provide additional insights into the translational utility of slice models systems. Furthermore, given the growing importance of targeted therapies and the limited access to human specimens receiving treatment, it will be useful to utilize human tumor slices to determine how these therapies impact the pathobiology of pancreas cancer.

Tumor-on-a-chip models present another ex vivo alternative. These models have been used for the study of pancreas cancer metastasis (72, 73), as well as to investigate the interactions between cancer cells and fibroblasts and their response to gemcitabine treatment (74). These models can be leveraged for drug screening and targeted therapy approaches (75, 76).

An understanding of how distinct conditions, such as nutrient availability (serum, glucose, amino acids), affect tumor biology and response to therapy in the ex vivo models is still largely lacking. Most ex vivo studies are performed at high glucose levels (16 mmol/L), equivalent to human hyperglycemic conditions (288 mg/dL), as well as with neutral pH (7.4), even though hypoxia is a key feature of the pancreas cancer TME. The composition of the culture medium largely affects gene expression (77) and is expected to alter the metabolism and overall biology of ex vivo models. The use of physiological cell culture medium that is representative of human plasma, such as human plasma–like medium or Plasmax (CancerTools) (78, 79), has the potential to improve the translation of findings from the ex vivo models to the clinic. Because of the reduced nutrient levels in plasma-like medium, medium must be replaced frequently or continuously refreshed. The nutrient availability and composition of tumor interstitial fluid (TIF) are also substantially different compared with those of plasma (80). TIF composition affects the interactions between stromal and tumor cells (81), as well as the metabolism of tumor cells (82). As such, the use of TIF supplementation can improve the use of ex vivo models in precision medicine approaches.

While tumor slices and tumor-on-a-chip models are highly useful biological tools, critical limitations of the models exist at the technical and biological levels. A current technical limitation is that these models are a limited resource and are non-renewable. Therefore, all experimental studies must be performed in a acute time window. Moreover, ex vivo slice cultures and tumor-on-a-chip models lack standardization, which jeopardizes reproducibility and translatability of findings. Furthermore, high-throughput drug screening is challenging in these models, either because of the limited material availability in the case of ex vivo slices or because of the lack of biocompatible materials in tumor-on-a-chip models. For ex vivo slices, communication between the staff providing the tumor specimen after surgical resection (e.g., biorepository) and the research group is critical to experimental success. Here, patient history of prior treatment, mutational analysis (if available), site of tissue resection (primary versus metastasis), and response to therapy (tumor imaging or biochemical response) are all critical variables that are essential to be incorporated into experimental planning and analysis. Further, specimen availability is limited to patients eligible for surgical resection, who account for less than 15% of all patients (7), limiting the utility of these approaches for individuals with late-stage disease. Moreover, slices have yet to be established from neoadjuvant-treated pancreas cancer patients after tumor resection.

There is potential for the use of ex vivo models in precision medicine approaches, such as to determine drug responses, but the technical limitations outlined above make widespread implementation of these methods difficult. Further studies investigating the optimal culture conditions, molecular characterization, and translatability into clinical outcomes are required for utilization of these models in precision medicine.

## In vivo models

While ex vivo cell culture models provide valuable insights into the cellular biology of pancreas cancer, mouse models are essential for understanding the complex in vivo dynamics of tumor progression. These models aim to recapitulate the genetic, histopathological, and clinical features of human pancreas cancer along with the numerous disease syndromes that complicate treatment, such as cachexia, malignant ascites, perineural invasion, and duodenal and bile duct blockage. Among the most frequently used mouse models of pancreas cancer are subcutaneous and orthotopic transplantation models (83) and genetically engineered mouse models (84–86).

### Subcutaneous and orthotopic models.

Subcutaneous models are generated by implantation of patient- or mouse-derived pancreas cancer cells or intact tumor pieces under the skin in the mouse flank. These models are relatively cost-effective and fast to establish, allowing for high-throughput screens of several treatments in a large number of animals. A model of patient-derived pancreatic tumor pieces subcutaneously grafted into immunocompromised mice has been used to assess the concordance in gemcitabine response between the patients and their matched xenografts (87), as well as in an in vivo drug screening platform (88). Subcutaneous models have also been used to evaluate the effects of novel or standard-of-care therapies on tumor burden and the TME (89, 90). These models have also successfully predicted responses to novel RAS inhibitors, revealing the importance of T cells for durable RAS inhibitor responses; have been used to evaluate the efficacy of RAS inhibitors with other targeted or standard-of-care therapies (31, 91); and have led to promising clinical trials (ClinicalTrials.gov NCT05379985). However, subcutaneous models bear little histological resemblance to human pancreas cancer. The subcutaneous TME is notably distinct from the native pancreatic TME, including differences in vascularization, fibrosis, and oxygen availability. As a result, tumor-stroma and tumor-immune interactions are different in these models compared with those in patients. These limitations may partly explain the higher RAS inhibitor response rate of the mouse subcutaneous transplantation models relative to the results of clinical trials (64% vs 39%, respectively). Moreover, these models do not reproduce the metastatic dissemination routes of pancreas cancer (92).

**Figure 2:**
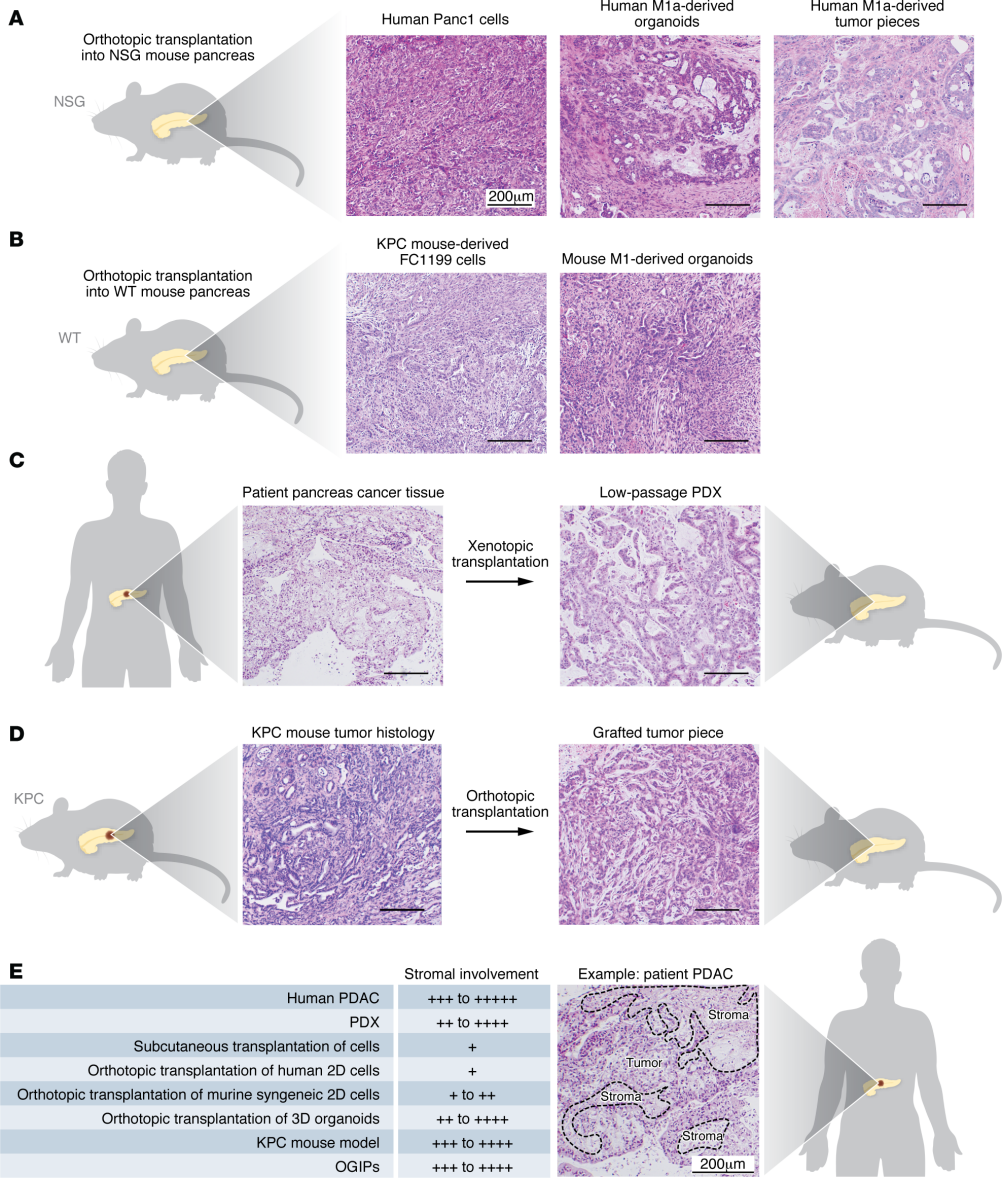
In vivo models of pancreas cancer. (**A**) Representative histology of pancreatic tumors from human Panc-1 2D pancreatic cancer cells, human M1a pancreatic cancer organoids, and intact pancreatic tumor pieces from human M1a organoid–derived tumors (orthotopically grafted intact pieces [OGIPs]) that were orthotopically transplanted into immunodeficient NOD/scid-gamma (NSG) mice. (**B**) Representative histology of pancreatic tumors from the KPC-derived FC1199 2D cell line and mouse M1 organoids that were orthotopically transplanted in the pancreas of syngeneic (C57BL/6J) mice. (**C**) Representative histology of human pancreas cancer tissue and its corresponding low-passage patient-derived xenograft (PDX). (**D**) Histology of a KPC tumor and its corresponding OGIP. (**E**) Comparison of stromal involvement in in vivo pancreatic cancer models, and patient pancreatic cancer image annotated as an example. PDAC, pancreatic ductal adenocarcinoma. All human specimens were deidentified and obtained from the UCSD Biorepository and Tissue Technology Shared Resource with approval from its Institutional Review Board. Written and informed consent was obtained prior to acquisition of tissue from all patients. Scale bars: 200 μm.

Orthotopic models are derived by implantation of patient- or mouse-derived pancreas cancer cells or intact tumor pieces directly into the mouse pancreas (Figure 2, A–C). These models more accurately recapitulate the native TME (Figure 2E) and capture the crosstalk between malignant and TME cells. In addition, orthotopic models enable the study of metastasis to distant organs, such as the liver (92), which provides insights into disease progression and treatment response. While physiologically relevant, orthotopic models are technically more challenging and require surgical expertise for laparotomy and tumor implantation. Tumor growth is often monitored using advanced imaging modalities such as bioluminescence or ultrasound, further increasing the cost and time required for orthotopic models.

### Patient-derived xenografts.

Patient-derived xenografts (PDXs) recapitulate inter- and intrapatient variability. These models are developed by either orthotopic or subcutaneous grafting of human tumor tissue directly into immunodeficient mice and then passaging of the tumor in vivo over time (93) (Figure 2C). Studies comparing biology and drug responses suggest some advantage to the orthotopic site, though it is technically more involved to establish, maintain, and monitor orthotopic tumors during preclinical therapeutic studies (94). To establish PDXs, multiple different immunodeficient strains have been used, a discussion that is beyond the scope of this Review. The most used strain to date is NOD/SCID-gamma, which is highly permissive to human tumor growth given the multiple defects in immune function. Murine stroma rapidly replaces human stromal cells, however, and given the immunodeficient background and lack of ligand/receptor signaling between species (e.g., HGF and MET), PDXs are not the model of choice to study many features of the TME (Figure 2, C and E). Further, the lack of a functioning immune system can lead to an underestimation of the impact of immunosuppression on pancreas cancer progression and treatment response.

Humanized mice can overcome this limitation of PDX models. Engrafting human immune cells in immunodeficient hosts reconstitutes the human immune system in mice and allows for the evaluation of immunotherapies in PDX models (95, 96). However, humanized PDX models are challenging to establish, show variability in the degree of reconstitution of the immune system, and often lead to graft-versus-host reactions. As such, humanized mice are not commonly used in PDX model development.

The clear advantage of PDXs is that the epithelial tumor cells accurately reflect the genome of the individual patient from whom they were derived. As whole-tissue xenografts, PDXs are also replete with an intact TME that resembles its founder tissue histology through early passages (Figure 2, C and E). PDXs are an excellent model to study biological features of the neoplastic cells and, in particular, novel therapies targeting oncogenic driver events. Many prior investigations have interrogated PDXs to understand their value in predicting response to chemotherapeutic agents (88, 97). Recently, PDXs were used to interrogate the pancreas cancer kinome and to identify potential therapeutic vulnerabilities (98). Another recent notable example of the utility of PDXs comes from studies of resistance to novel KRAS inhibitors and how this resistance correlates to the classical versus basal subtype (99). In general, these studies have demonstrated that PDXs have predictive utility, but the profound differences in drug delivery and the TME between these models and human pancreas cancer, as well as their cost and time consumption, detract from their ability to be widely deployed in precision medicine approaches (93).

### Genetically engineered mouse models.

An alternative to PDXs are autochthonous genetically engineered mouse models (GEMMs), which have been essential for understanding the complex dynamics of tumor progression. GEMMs allow for the study of pancreas cancer within its native microenvironment, enabling the observation of interactions between tumor cells, the immune system, and surrounding tissues. To date, GEMMs vary in the targeted genes, promoters, and/or Cre induction method (i.e., tamoxifen-inducible, exogenous Cre injection, etc.), with benefits and limitations to each. Key genetic alterations driving pancreas cancer, including mutations in *KRAS*, *TP53*, *CDKN2A*, and *SMAD4*, are incorporated into various GEMMs to model the disease initiation and progression (84–86).

Mutations in the *KRAS* oncogene are present in over 90% of pancreas cancer cases (100). The *Kras^G12D^* allele, a mutation that renders KRAS signaling constitutively active, is commonly paired with pancreas-targeted Cre-lox recombination systems using the *Pdx1* or *Ptf1a* promoter (101). This combination drives the development of pancreatic intraepithelial neoplasia (PanIN) resembling early human pancreas cancer. However, these models progress to invasive carcinoma with a long latency in the absence of additional genetic alterations, highlighting the need for more complex models to study advanced disease (101). Models expressing a doxycycline-induced *Kras* (i*Kras*) allow for more synchronous tumor growth, which enables the study of KRAS-dependent metabolic (102) and immune (103, 104) changes.

*TP53*, another tumor suppressor gene, is mutated in approximately 70% of pancreas cancer cases. Combining *Kras^G12D^* with conditional deletion or mutation of *Tp53* creates an accelerated system to study pancreas cancer progression. Interestingly, mouse models harboring mutations or deletions of *Tp53* are not functionally interchangeable, as they differ in the extent of tumor development, exocrine insufficiency, and metastatic potential (105). The *Pdx1*-*Cre* *LSL*-*Kras^G12D^* *Tp53^R172H/+^* (KPC) model accelerates the development of invasive pancreas cancer, mimicking human disease dynamics (106) (Figure 2, D and E). These mice display PanINs that rapidly progress to invasive adenocarcinoma, followed by systemic metastasis to sites including the liver and lungs (106). The KPC mouse model has proven invaluable in investigating tumor biology, including the mechanisms of tumor initiation, epithelial-mesenchymal transition, immune evasion, and therapeutic resistance. Although the KPC model is extensively used in pancreas cancer research, it does not fully recapitulate the mutational burden that is found in human pancreas cancer specimens (107, 108). Moreover, KPC mice develop spontaneous tumors, which complicates the management of treatment cohorts. One way to expedite this and still take full advantage of the KPC mouse model is to generate tumor pieces from KPC tumors and orthotopically graft them into the pancreas of wild-type mice. This allows for more synchronous tumor growth, with tumors that greatly resemble the histology of the donor tumors in terms of the stroma composition (109) (Figure 2, A, D, and E). These distinctions are crucial in understanding how different *Tp53* alterations influence pancreatic cancer biology. Another important caveat to these GEMMs is that all non-tumor cells are heterozygous for *Kras* and *Tp53*, which may limit translatability to human disease. These considerations are important to keep in mind when drawing conclusions about TME contributions in GEMMs. Notably, to address the contribution of wild-type *Tp53* in the TME, the Lozano laboratory has developed a *Tp53^wm-R172H/+^* model of breast cancer in which an exogenous *Tp53* cDNA is expressed in non-Cre-expressing cells (110). However, similar strategies have not yet been deployed for pancreas cancer.

Apart from *Tp53*, several other tumor suppressor genes have been used to model pancreas cancer in vivo. Conditional deletion of *Cdkn2a*, which encodes the frequently inactivated tumor suppressors p16INK4a and p19ARF, in *Kras^G12D^* mice enhances PanIN progression and invasive cancer development. The *Pdx1-Cre* *LSL-Kras^G12D^* *Cdkn2a^lox/lox^* model demonstrates accelerated tumorigenesis, emphasizing the cooperative effect of *Cdkn2a* loss and *Kras* activation (111). The loss of SMAD4, a critical mediator of TGF-β signaling, which occurs in nearly 50% of pancreas cancer cases, has also been modeled in vivo. GEMMs incorporating *Smad4* deletion alongside *Kras^G12D^* expression, such as *Pdx1-Cre* *LSL-Kras^G12D^* *Smad4^lox/lox^*, exhibit robust tumor progression and desmoplastic stromal responses, hallmarks of human pancreas cancer (112, 113).

Mouse models of pancreas cancer have been extensively used to study the efficacy of standard-of-care and novel therapies (30, 114). Resistance to MEK and PI3K or AKT inhibitors rapidly developed in the KPC mouse model, leading to only small increases in survival (115, 116), and recapitulating the lack of survival benefit and extreme toxicity in clinical trials (117, 118). Moreover, mouse models of pancreas cancer have been used to identify possible combinations of PARP inhibitors with PD-L1/PD-1 inhibitors (119), providing rationale for the design of novel clinical trials specifically for *BRCA*-mutant pancreas cancer patients. However, animal models are often limited in their translation to clinical outcomes. Among the most notable examples was the lack of improved clinical outcomes when hedgehog pathway inhibitors were combined with gemcitabine (120) or FOLFIRINOX (121), despite successful preclinical data generated using the KPC mouse model (122). Later work demonstrated that suppression of tumor stroma by genetic or pharmacological targeting of the hedgehog pathway leads to more aggressive and lethal tumors in animal models of pancreas cancer (123). This finding emphasizes the complexity of tumor and stroma interactions and suggests that the TME has both tumor-restraining and -promoting roles. Additionally, strategies that potentiate immunotherapy efficacy in GEMMs and transplantation models have also had insufficient translation to clinical outcomes due to the incomplete recapitulation of the immune microenvironment, which has been reviewed elsewhere previously (124, 125).

Chemoresistance is a big challenge in the treatment of pancreas cancer. Although resistance to chemotherapy drugs is well documented and extensively researched (126, 127), most preclinical studies are performed in a treatment-naive context. The lack of robust chemoresistant in vitro or in vivo models has several implications for clinical translation. Drug development on the basis of such oversimplified models fails to consider resistance mechanisms, leading to poor efficacy in clinical trials, which are often performed in patients with metastatic disease that progressed on chemotherapy. Development of models that better recapitulate the dynamic and heterogeneous nature of chemoresistant tumors will be critical for translational research to advance and improve outcomes in this difficult disease.

### In vivo models of metastatic disease.

An important drawback of the mouse models presented thus far is that the animals die as a result of primary disease burden. Most pancreas cancer patients present with metastasis at diagnosis (7). Even after resection of the primary tumor, distant metastases often occur, significantly affecting patient prognosis (128). Indeed, metastasis is the most common cause of cancer-related death. Therefore, understanding the mechanisms that drive distant metastasis, particularly to the liver, the most common site of pancreas cancer spread (129), is crucial to improve patient outcomes. Despite its importance, many of the in vivo and in vitro models that have been discussed so far do not capture the high degree of metastatic burden observed in most patients.

The KPC model is valuable for studying each step of the metastatic cascade, including invasion, intravasation, circulation, extravasation, and colonization (130). However, the timing of metastasis varies between individual mice, owing to the spontaneous nature of tumor formation. This poses challenges in obtaining consistent phenotypes across multiple animals (106). Moreover, as mentioned previously, the primary cause of death of KPC mice is primary tumor burden, whereas in human patients it is metastasis. This variability makes it challenging to study the role of specific genes during the metastatic process, as well as to evaluate therapeutic interventions. Orthotopic transplantation models using pancreas cancer cell lines present an alternative approach, but these models often result in rapid primary tumor growth with limited formation of distant metastases (131). To address this limitation, highly metastatic cell lines derived from specific metastases of orthotopic mouse models have been used to generate organ-specific metastases after subcutaneous or orthotopic injection in recipient mice and resection of the primary tumor (132). While this approach is particularly useful for recapitulating distant recurrence after primary tumor resection, a common clinical scenario, it requires the use of highly metastatic cell lines to form the primary tumors, which may not fully reflect the natural metastatic process, and it involves complicated and challenging surgical interventions in mice. Moreover, this method still exhibits relatively low spontaneous metastasis rates, with roughly 50% of the mice developing metastases (132). Despite its limitations, this model has been used to evaluate the effect of targeted therapies in combination with chemotherapy against metastases (32).

As the liver and, less frequently, the lungs are sites of distant metastasis in pancreas cancer patients (129), preclinical models of metastasis that target these organs are frequently employed. Liver metastasis models typically use hemi-spleen or portal vein injection techniques to mitigate the immune system disruptions that can occur with total splenectomy (133–135). To model lung metastases, tail vein injections are commonly performed. These models have been used to assess the impact of genetic perturbations or targeted therapies on metastatic burden (32, 136–138). However, tail vein injections can also induce liver metastases, depending on the cell line used (139). Intracardiac injection enables the systemic dissemination of cancer cells, allowing the formation of brain and bone metastases, but these are less common sites of metastasis in pancreatic cancer (140). Intralymphatic injection has also been reported as a model of lymph node metastasis (141), but it is not commonly employed in pancreatic cancer research because of the difficulty of reliably reproducing lymph node metastasis as it occurs in patients. However, spontaneous lymph node metastases are sometimes observed in orthotopic transplantation models. Given the limitations of current in vivo models of metastasis, early metastatic steps such as invasion and circulation are often examined in vitro. Assays such as invasion, migration, and ultra-low-attachment assays are commonly used to complement in vivo models (136, 137).

**Figure 3:**
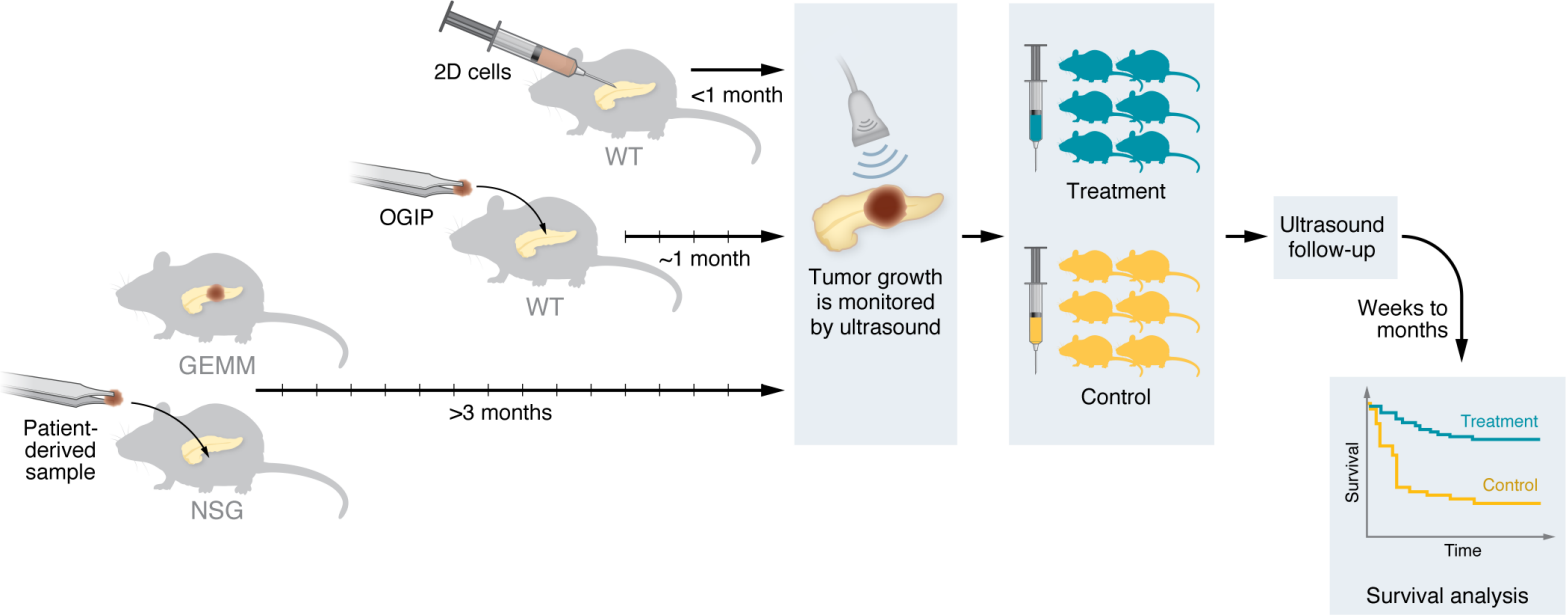
Example of animal hospital treatment study. Tumor formation varies from a few weeks (2D cell-derived models, OGIPs) to several months (GEMMs, PDXs in NSG mice). Tumor growth is monitored by ultrasound, and animals are enrolled in treatment groups. Tumor volume is evaluated by ultrasound throughout the study. A survival analysis is performed after several weeks or months of treatment.

Overall, in vivo models have greatly improved our understanding of pancreas cancer. The results of preclinical treatment studies have identified potential new therapies (Figure 3), which were later tested in clinical trials. Conversely, clinical trials have supplied preclinical researchers with patient samples that have been used in seminal work, such as the characterization of pancreas cancer molecular subtypes (10, 11). Moreover, clinical trials have evaluated treatment regimens, which are implemented in preclinical study designs to improve their translatability.

## Challenges and future directions

As already discussed throughout this Review, pancreas cancer is a complex disease, and patients have limited treatment options. Unlike other cancer types, such as non–small cell lung cancer, breast cancer, and melanoma, in which treatment strategies are often informed by the patients’ molecular subtype, precision medicine is yet not widely applied to patients with pancreas cancer. This is in part due to the lack of targeted therapies against the four most frequently mutated genes (*KRAS*, *TP53*, *SMAD4*, and *CDKN2A*) (142). PARP and KRAS^G12C^ inhibitors, as well as immunotherapy, have shown efficacy in the few pancreas cancer patients harboring *BRCA* mutations, *KRAS^G12C^* mutations, or microsatellite instability, respectively (143–145). However, these alterations are rare, with *BRCA* mutations found in 4%–5% of patients (146) and *KRAS^G12C^* mutations or microsatellite instability found in 1%–2% of patients (147, 148). Mutations in druggable signaling pathways are infrequent in patients with pancreas cancer (11). The distinct molecular subtypes of pancreas cancer are associated with treatment response but have not led to substantial changes in treatment approaches for patients (12, 13).

Fortunately, preclinical models have substantially improved our understanding of pancreas cancer biology and response to treatment. Current therapeutic challenges can be mitigated by implementation of lessons from these models. For instance, implementing molecular subtyping and identifying which patients have hot or cold immune microenvironments at enrollment but also during treatment or following progression may help inform treatment decisions and improve patient outcomes. However, the use of biomarkers is currently limited, possibly because of the complexity of pancreas cancer as well as the lack of standardized biomarkers. Overall, the characterization of predictive biomarkers for pancreas cancer requires a combinatorial approach that will incorporate genetic, molecular, immune, and other factors to appropriately describe the heterogeneity of pancreas cancer patients.

In recent decades, the 5-year survival of pancreas cancer patients has shown a significant improvement from 7% in the early 2000s to 13% in 2024 (2, 149). Yet pancreas cancer remains one of the most lethal malignancies. The advanced stage at diagnosis, the intrinsic resistance of pancreas cancer lesions, and the lack of therapies targeting the driving oncogenes contribute to the difficulty of curing this disease. Although the development of the novel RAS inhibitors is expected to revolutionize patient care in the coming years, combination therapies are still expected to be necessary for the successful management of the disease. In this context, patients will greatly benefit from precision medicine approaches. PDOs, GEMMs, and PDXs leverage substantial strengths and, together, mitigate the weaknesses of each individual model system, providing platforms for the rapid screening of drug combinations, in the setting of either primary, relapse, or metastatic disease. The strengths of these models can be leveraged by artificial intelligence and machine learning approaches, which can improve biomarker and drug discovery but also can infer human relevance from preclinical studies (150–152).

The main limitation on the application of the patient-derived models presented in this Review in precision medicine is the time from tissue isolation to model establishment (Figures 1 and 3). However, PDOs can be generated and used for drug screens that can inform patient care within 7–15 days after tumor resection (61), whereas PDX models require several months of in vivo expansion before enough animals are available for use in treatment studies. As such, PDOs can be useful in predicting patient responses in precision medicine approaches. As technologies improve and knowledge for the establishment of pancreas cancer models is disseminated within the research and clinical community, these models will become integrated in routine clinical practice and can lead to improved patient outcomes.

## Conclusion

Experimental models have played a critical role in advancing our understanding of pancreas cancer biology and identifying potential therapeutic strategies. While each model has its strengths and limitations, the integration of in vitro, ex vivo, and in vivo systems offers a comprehensive approach to studying this complex disease. As precision medicine gains traction, patient-derived models, such as PDOs and PDXs, will be invaluable for guiding personalized treatment strategies. By addressing the challenges of current models and leveraging emerging technologies, we can pave the way to more effective therapies and improved outcomes for pancreas cancer patients.

## Version 1 08/15/2025

Electronic publication
